# Simultaneous Alcoholic and Malolactic Fermentations by *Saccharomyces cerevisiae* and *Oenococcus oeni* Cells Co-immobilized in Alginate Beads

**DOI:** 10.3389/fmicb.2016.00943

**Published:** 2016-06-14

**Authors:** Gianluca Bleve, Maria Tufariello, Cosimo Vetrano, Giovanni Mita, Francesco Grieco

**Affiliations:** Unità Operativa di Lecce, Consiglio Nazionale delle Ricerche - Istituto di Scienze delle Produzioni AlimentariLecce, Italy

**Keywords:** wine fermentation, *Saccharomyces cerevisiae*, *Oenococcus oeni*, co-immobilization, biocatalyst for wine

## Abstract

Malolactic fermentation (MLF) usually takes place after the end of alcoholic fermentation (AF). However, the inoculation of lactic acid bacteria together with yeast starter cultures is a promising system to enhance the quality and safety of wine. In recent years, the use of immobilized cell systems has been investigated, with interesting results, for the production of different fermented foods and beverages. In this study we have carried out the simultaneous immobilization of *Saccharomyces cerevisiae* and *Oenococcus oeni* in alginate beads and used them in microvinifications tests to produce Negroamaro wine. The process was monitored by chemical and sensorial analyses and dominance of starters and cell leaking from beads were also checked. Co-immobilization of *S. cerevisiae* and *O. oeni* allowed to perform an efficient fermentation process, producing low volatile acidity levels and ethanol and glycerol concentrations comparable with those obtained by cell sequential inoculum and co-inoculum of yeast and bacteria cells in free form. More importantly, co-immobilization strategy produced a significant decrease of the time requested to complete AF and MLF. The immobilized cells could be efficiently reused for the wine fermentation at least three times without any apparent loss of cell metabolic activities. This integrated biocatalytic system is able to perform simultaneously AF and MLF, producing wines similar in organoleptic traits in comparison with wines fermented following traditional sequential AF and MLF with free cell starters. The immobilized-cell system, that we here describe for the first time in our knowledge, offers many advantages over conventional free cell fermentations, including: (i) elimination of non-productive cell growth phases; (ii) feasibility of continuous processing; (iii) re-use of the biocatalyst.

## Introduction

Yeasts are the most important microorganisms responsible of alcoholic fermentation (AF), whereas lactic acid bacteria are able to perform malolactic fermentation (MLF) in winemaking (Diviès and Cachon, 2005). Several yeast commercial starter cultures are nowadays available for production of safe wines improved in desirable taste and aroma features (Romano et al., 2003). MLF is a secondary process that can spontaneously occur several weeks after the AF, during storage of young wines. In fact, it consists of conversion, promoted by malolactic bacteria, of L-malic acid into L-lactic acid and CO_2_, it is responsible of the acidity reduction and of the pH increase and it can contribute to the final wine flavor complexity (Bauer and Dicks, 2004). However, this bioprocess is unpredictable and very slow. Although starters have been selected also for the MLF, their use did not ensure that the process will start, occur, or be completed, especially due to the unfavorable conditions of the wine-environment for the bacterial growth (Alexandre et al., 2004).

In recent years, it is increasing the interest in using immobilized cells for fermentation processes, such as beer production (Masschelein et al., 1994), cider production (Nedovic et al., 2000), sparkling wine fermentation (Yokotsuka et al., 1997). Compared to the conventional free cell system, these strategies offer numerous technical and economic advantages (Nedović et al., 2011). Several immobilization systems have been studied for wine fermentation, such as calcium and sodium alginate, delignified cellulosic materials, Kissiris, DEAE–cellulose (Kosseva and Kennedy, 2004; Kourkoutas et al., 2004; Agouridis et al., 2008) and starchy materials (Nedović et al., 2015).

Immobilization approaches can influence yeast and bacteria metabolism producing effects on wine quality, aroma, and taste. It has also been demonstrated that these systems can improve AF and MLF productivity and economic efficiency, since immobilization can make easier to control the process and produces an acceleration of it (Melzoch et al., 1994; Sipsas et al., 2009; Vila-Crespo et al., 2010). Immobilization systems offer also the advantage to reuse the biocatalysts for several times without loss of fermentation activities, to perform continuous process and to decrease capital costs reducing bioreactor volumes (Pilkington et al., 1998; Bleve et al., 2011). It gives also the opportunity to co-immobilize different kind of microorganisms within the same porous matrix, allowing the accomplishment of the two fermentation steps in one integrated system.

In this study, for the first time in our knowledge, we have immobilized in a calcium alginate matrix a mixed AF/MLF starter i.e., a commercial strain of *S. cerevisiae* and a commercial strain of *O. oeni*. We have used this immobilized multistarter mix to promote the fermentation of Negroamaro must. The obtained wines were characterized for their fermentation kinetics, chemical profiles associated to AF and MLF and flavor profiles.

## Materials and methods

### Yeast, bacterial strains, and media

*Oenococcus oeni* strain Lalvin VP41™ and *Saccharomyces cerevisiae* strain Lalvin ICV-D254® were supplied by Lallemand Fermented Beverages (Italy). Growth medium for *O. oeni* strain and for the propagation in alginate beads was FT80 medium (Cavin et al., 1989). The bacteria were grown at 30°C for 2–3 days under anaerobic conditions. The initial pH-values were adjusted to 5.2. Cycloheximide was added at a concentration of 100 mg/L in the solid and liquid FT80 media, in order to selectively count bacteria in wine samples inoculated also with *S. cerevisiae*. Growth medium for *S. cerevisiae* strain and for the propagation in alginate beads was YPD at 30°C for 16–18 h. Ampicillin was added at a concentration of 50 mg/L in the solid and liquid YPD media, in order to selectively count yeasts in wine samples inoculated also with *O. oeni*. Solid media were prepared with addition of 2% (w/v) agar.

### Immobilization of *Oenococcus oeni* whole cells in ca-alginate gel beads

The following parameters of sodium alginate concentration and initial inoculum quantity were optimized for the preparation of beads. Different concentrations of sodium alginate (2, 3, and 4% w/v) were tested. The initial biomass to be packed in the beads was ascertained by incorporating in the aqueous sodium alginate solution different amount of cells corresponding to 10^4^, 10^5^, 10^6^, or 10^7^ CFU/mL. The *O. oeni* cells were grown in FT80 medium at 30°C for 24–48 h. When the turbidity of the culture reached an optical density at 600 nm = 0.8, the requested volume was harvested. Cells were washed with water and then suspended in Na-alginate (Sigma, USA) solution at the above indicated concentrations and then cured using a 0.1 M CaCl_2_ solution. The beads were prepared following the procedure described by Bleve et al. (2008). Beads were then washed with saline solution, added with 250 mL of sterilized must (sugars 173.4 ± 0.7, malic acid 2.25 ± 0.4, pH 3.33) and subjected to fermentation at room temperature.

Viable counts of cells were periodically evaluated on agar plates by spreading 10-fold serial dilutions of the fermented must onto FT80 added with cycloheximide at 30°C for 2–3 days under anaerobic conditions.

### Immobilization of *S. cerevisiae* and *O. oeni* whole cells in Ca-alginate gel beads

The yeast cells were grown in YPD medium with shaking at 25°C for 16 h, whereas *O. oeni* cells were grown in FT80 medium at 30°C for 24–48 h. When the turbidity of the yeast and bacteria cultures reached an optical density of 0.8 at 600 nm, the requested volume was harvested. Cells were washed with water and then suspended in aliquot 3% Na-alginate (Sigma, USA) solution to obtain a final concentration of 10^6^ CFU/mL for *O. oeni* and for *S. cerevisiae*. The beads were prepared following the procedure described by Bleve et al. (2008), using a 0.1 M CaCl_2_ solution. The spherical beads (ca. 3 mm diameter) produced were cured in 0.1 M CaCl_2_ solution for 4 h at 4°C.

### Micro-fermentation assays

Micro-fermentation assays were conducted in Negroamaro grape must, as previously described (Tristezza et al., 2012). The must was filtered twice both through cheesecloth and a 0.22-μm membrane filter and, then, it was combined with 50 mg/L potassium metabisulphite. The must composition was: reducing sugars 220 ± 0.6 g/L, Brix 19.7, total acidity 6.12 ± 0.7 g/L, volatile acidity 0.22 ± 0.02 g/L, pH 3.3, malic acid 4.5 ± 0.3 g/L, tartaric acid 2.5 ± 0.08 g/L, glycerol 0.68 ± 0.04 g/L, density 1.07 g/ml. Two hundred milliliters of must were placed in sterile Erlenmeyer 250-mL flasks and then inoculated with yeast and bacteria in free and immobilized form pre-cultured in specific media described above. The relative volume of beads inoculated in 200 ml of grape must was about 35–40 ml, corresponding to an additional 17.5–20% of the final volume. Alcoholic fermentations were carried out at 25°C and samples were weighted daily in order to follow the volatile CO_2_ production until the weight was constant.

MLF was monitored at determined time intervals by the depletion of L-malic acid and this organic acid was detected using an enzymatic kit (La Roche, Basel, Switzerland). Each fermentation experiment was carried out by performing three simultaneous and independent tests.

An aliquot of fermented must (100 mL) was stored at −20°C, whereas the remaining wine was used for sensorial analysis. Each fermentation experiment was carried out by performing three simultaneous and independent tests.

The experimental plan included 10 trials based on different combinations of inocula:

- beads containing co-immobilized *S. cerevisiae* and *O. oeni* cells, corresponding to an inoculum equivalent to 10^6^ CFU/mL of *O. oeni* and of *S. cerevisiae* (sample C1);
- a simultaneous inoculum of beads containing *S. cerevisiae* and *O. oeni* cells separately immobilized, corresponding to an inoculum equivalent to 10^6^ CFU/mL of *O. oeni* or of *S. cerevisiae* (sample C2);
- a simultaneous inoculum of beads contai*n*ing *S. cerevisiae* corresponding to an inoculum equivalent to 10^6^ CFU/mL and an inoculum of free 10^6^ CFU/mL *O. oeni* cells (sample C3);
- beads containing an inoculum of 10^6^ CFU/mL *S. cerevisiae* cells, followed, at the end of AF, by beads containing an inoculum equivalent to 10^6^ CFU/mL of *O. oeni* (sample C4);
- a simultaneous inoculum of 10^6^ CFU/mL free *S. cerevisiae* and 10^6^ CFU/mL *O. oeni* cells (sample C5);
- an inoculum of free 10^6^ CFU/mL *S. cerevisiae* cells, followed, at the end of AF, by an inoculum equivalent to 10^6^ CFU/mL free cells of *O. oeni* (sample C6);
- an inoculum of free 10^6^ CFU/mL *S. cerevisiae* cells, followed, at the end of AF, by beads containing an inoculum equivalent to 10^6^ CFU/mL of *O. oeni* (sample C7);
- an inoculum of free 10^6^ CFU/mL *S. cerevisiae* cells to perform the only AF (sample C8);
- control sample of must treated with uninoculated beads (sample C9);
- uninoculated must control sample (sample C10).

Viable counts of cells were periodically evaluated on agar plates by spreading 10-fold serial dilutions of the fermented must onto YPD agar added with ampicillin and incubating at 28°C overnight for enumeration of yeasts and onto FT80 added with cycloheximide at 30°C for 2–3 days under anaerobic conditions for enumeration of oenococci. Also samples inoculated with free cells of *S. cerevisiae* and/or *O. oeni* were screened in the same conditions.

### Chemical analysis

General wine parameters (alcohol content, residual sugars, pH, titratable and volatile acidity, tartaric, citric, lactic acid, glycerol, and total sulfur dioxide) were determined using WineScan FT120 (Foss, Hillerød, Denmark) instrument. Malic acid was detected using an enzymatic kit (La Roche, Basel, Switzerland). The analyses were performed in triplicate.

### Reagents and standards

The standards of volatile compounds were purchased from Sigma–Aldrich (St. Louis, MO), with purity superior to 98%; methanol (HPLC gradient grade) and ethanol 96% were purchased from J.T. Baker (Phillipsburg, NJ, USA), and dichloromethane were purchased from Carlo Erba Reactive (Rodano, Italy). Pure water was obtained from a Milli-Q purification system (Millipore, USA).

### Extraction of volatile compounds

The extraction of volatile compounds was carried out with a solid phase extraction (SPE) procedure, using polymeric sorbents and dichloromethane as elution sorbent (Ferreira et al., 1995; Piñeiro et al., 2006; Capone et al., 2013). A Vac Elut 20 station equipment from Varian (Palo Alto, USA) was used. The wine aroma compounds were separated by adsorption/desorption on cartridges. Strata polymeric SPE sorbents (styrene-divinylbenzene) prepacked in 500 mg/6 mL cartridges (Phenomenex) were first rinsed with 4 mL of dichloromethane, 4 mL of methanol and, finally, 4 mL of a water–ethanol mixture (12%, v/v).

To 50 mL of each wine sample and to each standard solution were added 300 μL of internal standard solution (2-octanol; 200 mg/L hydro-alcoholic solution). Each liquid sample was passed through the SPE cartridge at around 2 mL/min. Afterwards, the sorbent was dried by letting air pass through it. The volatile compounds were recovered by elution with 4 mL of dichloromethane, and concentrated to a final volume of 500 μL under a stream of pure nitrogen (N_2_) (Shinohara, 1985; Vilanova and Sieiro, 2006; Gómez García-Carpintero et al., 2011a).

The sample (1 μL) was injected in the gas chromatographic system, the analyses were performed in triplicate and mean values were used in further data processing.

### GC-MS conditions and quantitative analysis

A 6890N series gas chromatograph (Agilent Technologies) with an Agilent 5973 mass spectrometer selective detector (MSD) and equipped with a DB-WAX capillary column (60 m^*^0.25 mm I.D., 0.25 μm film thickness, Agilent Technologies) was used. The carrier gas was helium at a flow rate of 1.0 mL/min. A split/splitless injector was used in the splitless mode, the injector temperature was 250°C and the injected volume was 2 μL. The column oven temperature was initially held at 40°C, then it was programmed to 200°C at 4°C/min, with a final holding time of 20 min. Spectra were recorded in the electron impact mode (ionization energy, 70 eV) in a range of 30–500 amu at 3.2 scans/s. A solvent delay time of 10 min was used to avoid overloading the mass spectrometer with solvent.

The identification of the volatile compounds was achieved by comparing mass spectra with those of the data system library (NIST 98, *P* > 90%), with the retention data of commercially available standards and MS data reported in the literature. Quantification analysis is based on the principle that the component area is proportional to the amount of the analyte present in the sample. The quantification was carried out following the internal standard quantification method.

### Odor activity value

The specific contribution of each odorant compound to the overall wine aroma was determined by calculating the odor activity value (OAV) as the ratio of the concentration of each compound to its detection threshold concentration (Francis and Newton, 2005). An odor profile for the wines was obtained by grouping the volatile compounds with similar descriptor in specific aromatic series. The value of each aromatic series was obtained adding the OAVs of the compounds that form such a series. Therefore, it is possible to determine the contribution of a specific compound to each series. This procedure makes to relate quantitative information obtained by chemical analysis (GC-MS) to sensory perception, providing a hybrid chemical/sensory fingerprinting (Capone et al., 2013).

### Statistical analysis

Statistical analysis of the wine general parameters and wine volatile concentrations were performed using an analysis of variance (ANOVA) to determine statistically different values at a significance level of *P* ≤ 0.05.

The comparison of volatile classes of compounds during fermentations was achieved by principal component analysis (PCA). All statistical analyses were carried out using the STATISTICA 7.0 software (StatSoft software package, Tulsa, OK, USA).

## Results

### Optimization of *Oenoccus oeni* immobilization in calcium alginate

**Figure 1:**
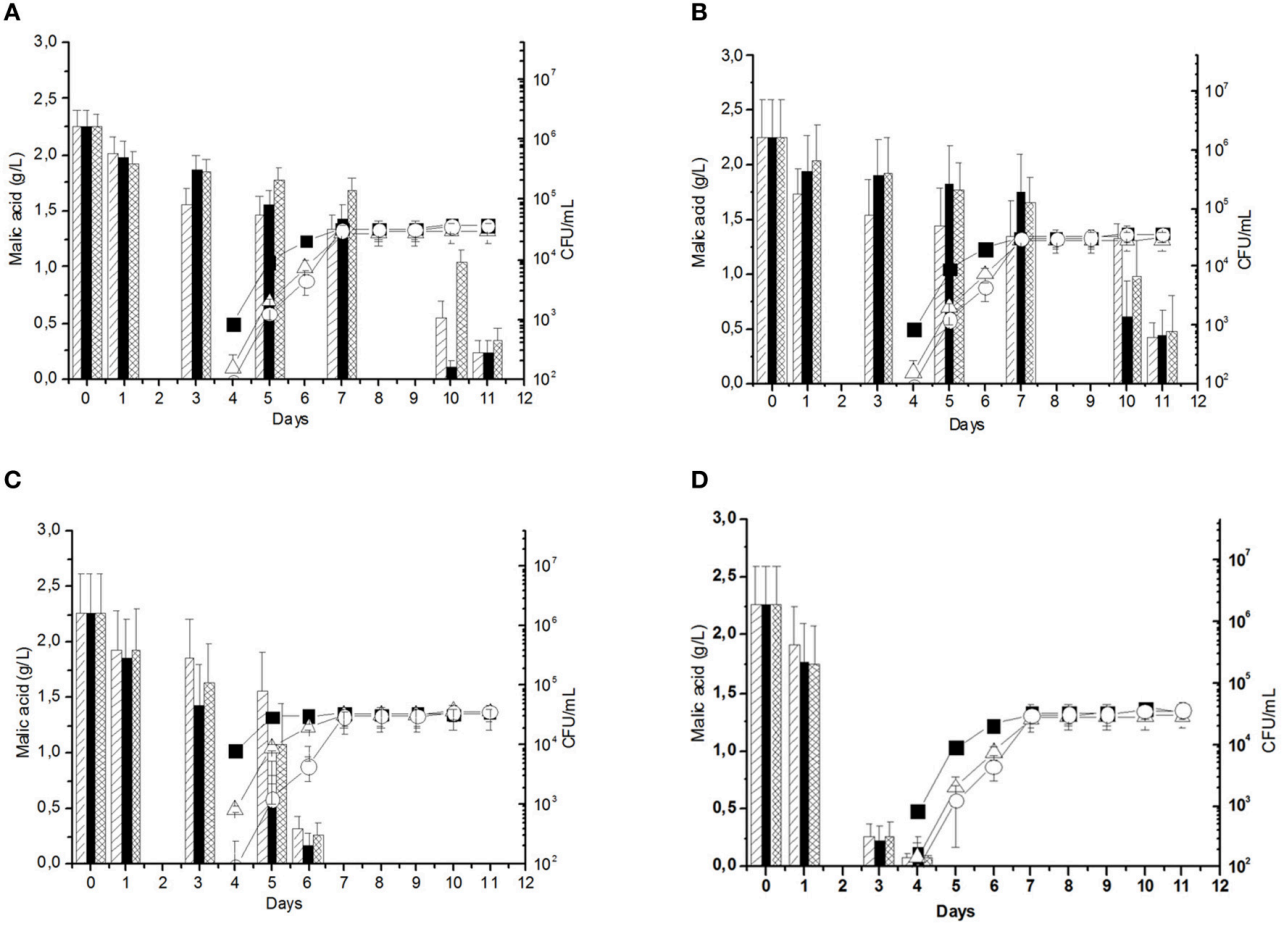
**Malic acid consumption in Negroamaro must inoculated with beads of 2% 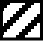, 3% 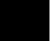, and 4% 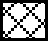 calcium alginate containing *Oenococcus oeni* strain Lalvin VP41™**. Panels **(A–D)** refer to results obtained using calcium alginate beads containing 10^4^, 10^5^, 10^6^, 10^7^ CFU/ml of *O. oeni*, respectively. Cell leaking and cell growth (CFU/ml) from beads containing 2% 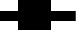, 3% 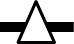, and 4% 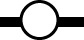 (w/v) calcium alginate is indicated in each panel. The curves indicate the cell leaking.

The first step of this study consisted in the optimization of the following parameters for the *O. oeni* the immobilization, i.e., initial cell biomass to be loaded in the beads and sodium alginate concentration, in order to improve bead properties, such as permeability and rigidity. Different *O. oeni* cell concentrations, i.e., 10^4^, 10^5^, 10^6^, and 10^7^ CFU/mL, were individually immobilized in each beads preparation using 2, 3, and 4% (w/v) sodium alginate concentrations. Malolactic fermentation experiments were performed for each sample in filtered *Negroamaro* must. As reported in Figure 1, malic acid was completely consumed after 6 and 4 days of fermentation when inocula corresponding to 10^6^ (Figure 1C) and 10^7^ (Figure 1D) CFU/mL were used, respectively. Instead, the use of lower cell concentrations (10^4^ and 10^5^ CFU/mL, Figures 1A,B) did not produce a complete consumption of malic acid along the 11 days period of the experiment. In the used experimental conditions, comparable cell counts were recorded in all the analyzed samples, thus suggesting that the immobilization conditions and initial cell inocula did not influence final cell counts into the medium. The use of 3% (w/v) calcium alginate and of an inoculum of *O. oeni* cells corresponding to 10^6^ CFU/mL resulted the minimum conditions to obtain a significant reduction in time required to completely reduce malic acid. In addition, the use of 3% (w/v) calcium alginate was suitable to perform co-immobilization experiments with yeasts, since it resulted the best conditions to immobilize yeasts (Bleve et al., 2008).

### Microfermentations using different yeast and bacteria inoculation strategies

**Figure 2:**
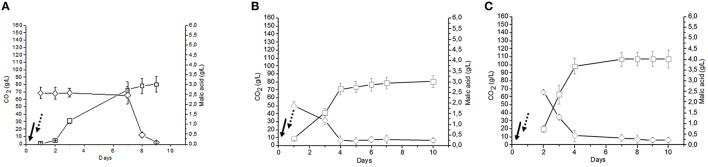
**Cycles of Negroamaro must fermentations inoculated with *Oenococcus oeni* strain Lalvin VP41™ and *Saccharomyces cerevisiae* strain Lalvin ICV-D254® co-immobilized in alginate beads (sample C1)**. Each fermentation cycle was performed for 10 days. At the end of AF and MLF, the fermentation cycle was considered completed, the wine was removed and submitted to quality assays. The beads were collected and, after a wash with sterile saline solution (0.9% NaCl), reinoculated for another fermentation cycle. Letters on the top of each panel indicate the three different cycles of fermentation **(A–C)**. CO_2_ (g/l) production 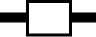, Malic acid consumption 
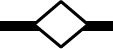
. Inoculation time of *S. cerevisiae*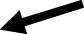, inoculation time of *O. oeni*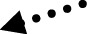.

**Figure 3:**
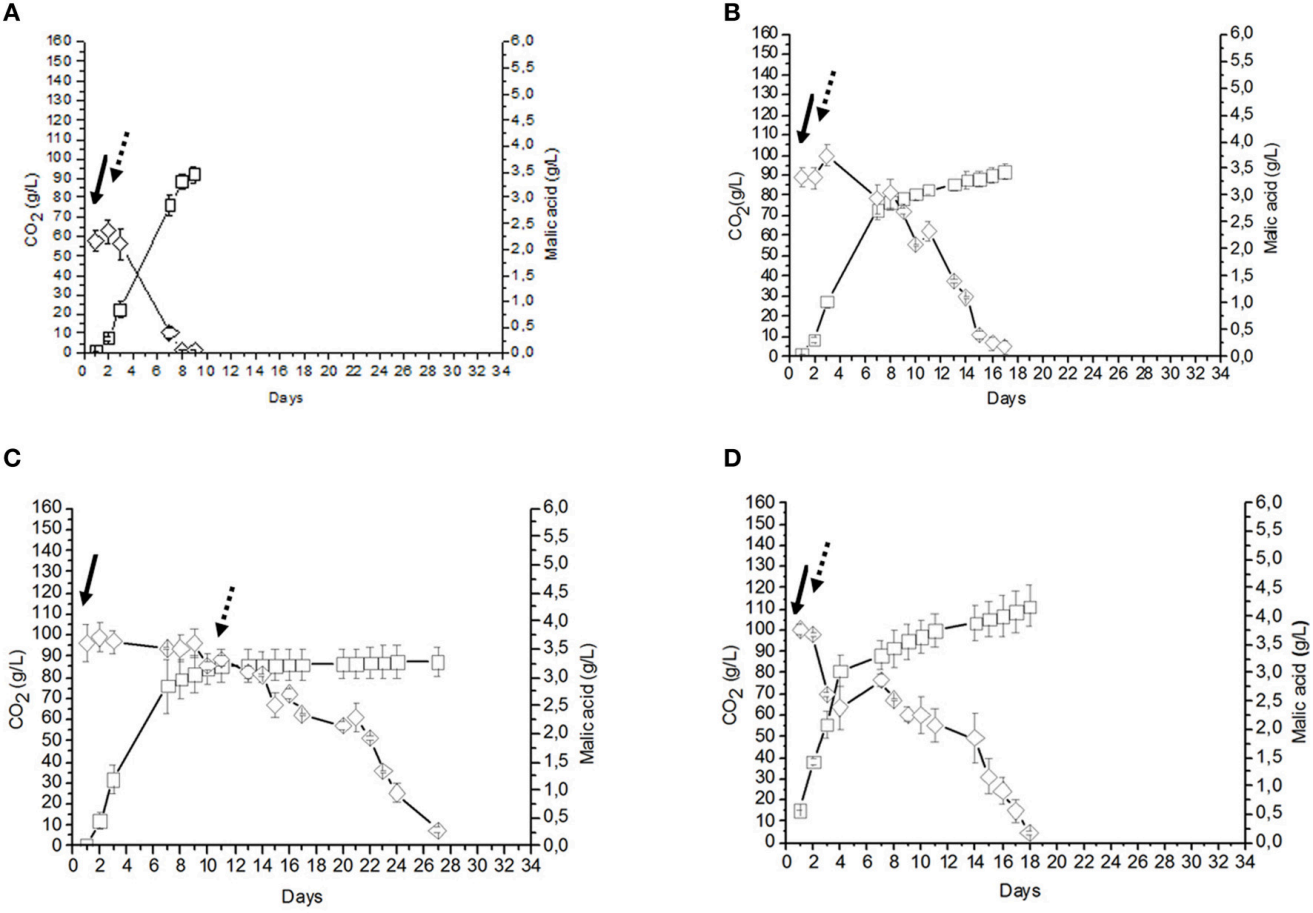
**(A)** Must fermentation performed by simultaneous inoculum of beads containing *S. cerevisiae* and *O. oeni* cells separately immobilized, (sample C2). **(B)** Must fermentation performed by simultaneous inoculum of beads containing *S. cerevisiae* and an inoculum of free (10^6^ CFU/ml) *O. oeni* cells (sample C3). **(C)** Fermentation using beads containing *S. cerevisiae* cells, followed, at the end of AF, by beads containing *O. oeni* (sample C4). **(D)** Fermentation performed by using simultaneous inoculum of (10^6^ CFU/ml) free *S. cerevisiae* and (10^6^ CFU/ml) *O. oeni* cells (sample C5); CO2 (g/l) production 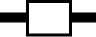, Malic acid consumption
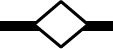
. Inoculation time of *S. cerevisiae* 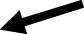, inoculation time of *O. oeni*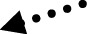.

**Figure 4:**
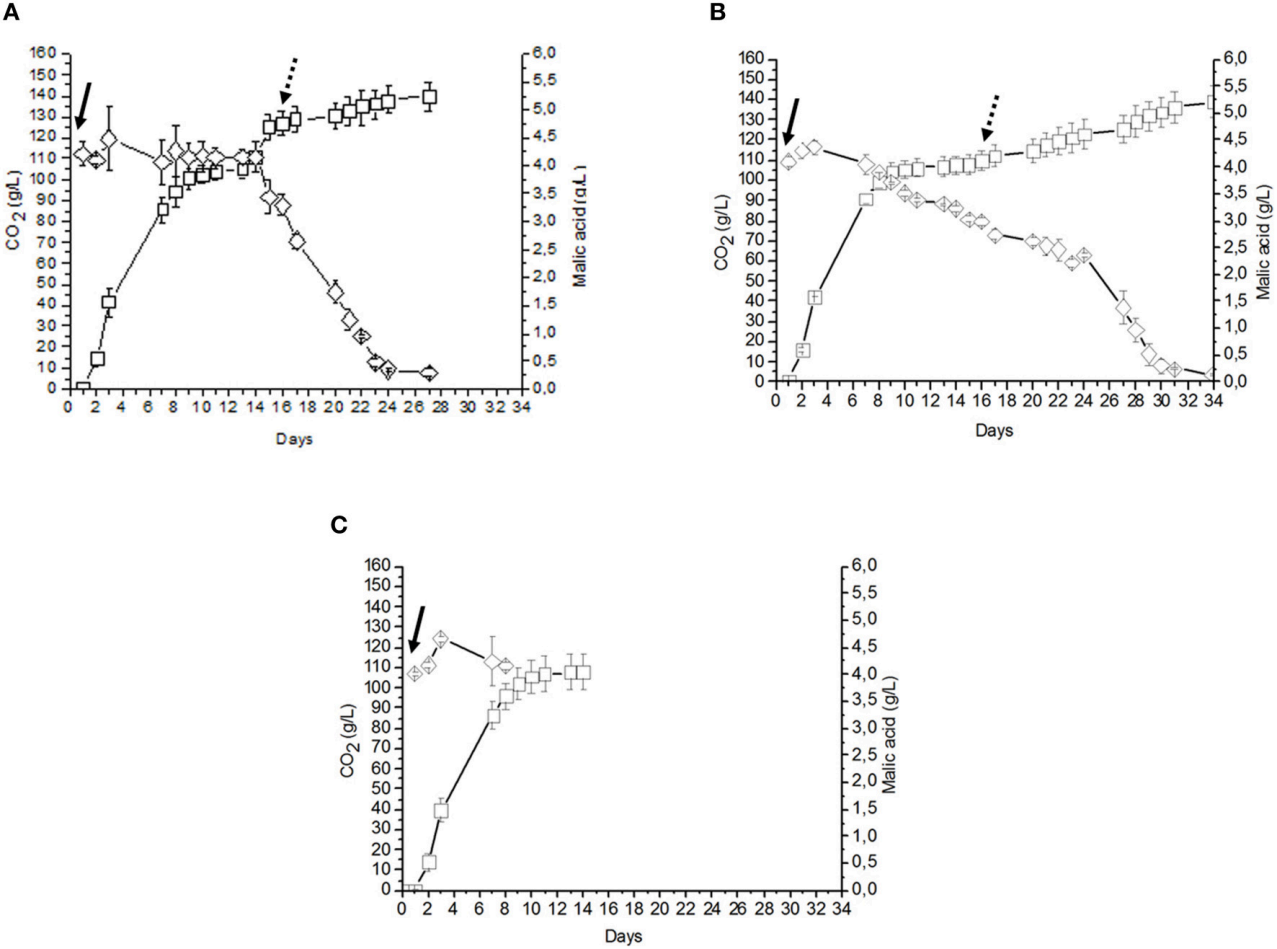
**(A)** Fermentation performed using free (10^6^ CFU/ml) *S. cerevisiae* cells, followed, at the end of AF, by free cells (10^6^ CFU/ml) of *O. oeni* (sample C6). **(B)** Fermentation performed by free (10^6^ CFU/ml) *S. cerevisiae* cells, followed, at the end of AF, by beads containing *O. oeni* (sample C7). **(C)** AF performed by an inoculum of free 10^6^ CFU/ml *S. cerevisiae* cells (sample C8). CO_2_ (g/l) production 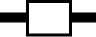, Malic acid consumption
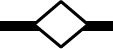
. Inoculation time of *S. cerevisiae* 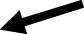, inoculation time of *O. oeni*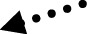.

The fermentative performances of the two immobilization strategies, i.e., co-immobilized *S. cerevisiae* and *O. oeni* (C1 sample) and co-inoculation of beads containing separately immobilized *S. cerevisiae* and *O. oeni* (C2 sample) were analyzed. For both the utilized strategies, AF and MLF occurred simultaneously and resulted in a significant shortening of the time requested to complete the fermentation. In fact, the process was completed in a maximum of 10 days (Figures 2A, 3A). The cell counts deriving from cell leakage from beads and their simultaneous growth in liquid ranged from 1.85 to12.5 × 10^6^ CFU/ml for *S. cerevisiae* and from 0.012 to 0.036 × 10^6^ CFU/ml for *O. oeni* in C1 and C2 samples. Moreover, the co-immobilized *S. cerevisiae* and *O. oeni* cells (C1 sample) was efficiently reused for the wine fermentation at least three times without any apparent loss of cell metabolic activities and cell viability (Figures 2B,C). As expected, the sequential inoculum of beads containing separately immobilized *S. cerevisiae* and *O. oeni* (C4 sample) produced a significant increase in time needed to obtain the end of MLF (28 days), whereas the AF was completed after 10 days (Figure 3C). Since the addition of beads (volume corresponding to about 35–40 ml) to grape must (200 ml) produced and increase in the final volume of about 15–20%, the use of beads to immobilize microorganisms reduced to about 80% of some initial metabolites (sugars, total acidity, malic acid, citric acid, glycerol, total SO2) concentrations (C9), compared to uninoculated control (C10) (Table 1). The AF and MLF processes, carried out following the traditional sequential inoculation strategy (C6 sample), were completed after a period of 30 days (Figure 4A).

**Table 1:** **Physicochemical parameters of wine samples**.

**Parameters**	**Wines**									
	**C1**	**C2**	**C3**	**C4**	**C5**	**C6**	**C7**	**C8**	**C9**	**C10**
Alcoholic degree (%vol)	8.91^e^±0.21	7.90^c^±0.05	8.4^d^±0.01	7.14^b^±0	10.58^g^±0.02	11.32^h^±0.01	9.53^f^±0.31	11.35^h^±0.11	0.41*a*±0.01	0.20^a^±0.04
Reducing sugar (g/L)	1.66^a^ ± 0.08	1.29^a^ ± 0.01	1.61^a^ ± 0.04	1.01^a^ ± 0.03	2.46^a^ ± 0.04	2.28^a^ ± 0.02	1.93^a^ ± 0	2.41^a^ ± 0.01	165.85b ± 3.05	214.34^c^ ± 4.05
Total acidity (g/L)	4.24^b^ ± 0.08	3.81^a^ ± 0.02	4.30^b^ ± 0.01	3.90^a^ ± 0.02	5.32^c^ ± 0.02	5.48^d^ ± 0.01	5.06^c^ ± 0.03	7.18^f^ ± 0.02	5.22^c^ ± 0.2	6.99^e^ ± 0.08
Volatile acidity (g/L)	0.57^d^ ± 0.02	0.52^c^ ± 0.01	0.56^d^ ± 0	0.60^e^ ± 0	0.55^d^ ± 0.01	0.50^c^ ± 0.01	0.53^c^ ± 0	0.44^b^ ± 0.01	0.20^a^ ± 0.01	0.23^a^ ± 0.02
pH	3.42^c^ ± 0	3.40^c^ ± 0	3.43^c^ ± 0.01	3.42^c^ ± 0	3.47^d^ ± 0	3.38^b^ ± 0.01	3.40^c^ ± 0.01	3.23^a^ ± 0.01	3.33^b^ ± 0.01	3.33^b^ ± 0.03
Lactic acid (g/L)	2.31^e^ ± 0.04	2.17^d^ ± 0.01	1.74^b^ ± 0.01	1.59^a^ ± 0.01	2.1^c^ ± 0	2.78^f^ ± 0.02	2.82^f^ ± 0.01	ND	ND	ND
Malic acid (g/L)	ND	ND	0.22^a^ ± 0.02	0.18^a^ ± 0.04	0.25^a^ ± 0.03	0.23^a^ ± 0.04	0.17^a^ ± 0.02	4.2^c^ ± 0.40	3.7^b^ ± 0.30	4.6^c^ ± 0.04
Tartaric acid (g/L)	2.01^d^ ± 0.03	2.04^d^ ± 0.01	1.87^c^ ± 0.01	1.63^b^ ± 0.02	1.42^a^ ± 0.01	1.79^c^ ± 0.02	1.79^c^ ± 0.01	1.73^c^ ± 0.03	1.52^b^ ± 0.06	2.39^e^ ± 0.09
Citric acid (g/L)	0.22^b^ ± 0.01	0.23^b^ ± 0.01	0.22^b^ ± 0.01	0.17^a^ ± 0	0.36^c^ ± 0	0.29^b^ ± 0.01	0.25^b^ ± 0	0.41^c^ ± 0	ND	ND
Density (g/mL)	1.00^a^ ± 0	1.00^a^ ± 0	1.00^a^ ± 0	1.00^a^ ± 0	1.00^a^ ± 0	0.99^a^ ± 0	0.99^a^ ± 0	0.99^a^ ± 0	1.06^b^ ± 0	1.08^b^ ± 0
Glycerol (g/L)	5.19^e^ ± 0.20	4.88^d^ ± 0.02	4.82^d^ ± 0.02	4.15^c^ ± 0.02	5.68^f^ ± 0.06	6.09^g^ ± 0.03	5.60^f^ ± 0.02	6.36^h^ ± 0	0.25^a^ ± 0.05	0.58^b^ ± 0.17
Total SO_2_(mg/L)	87.33^c^ ± 1.66	78.83^b^ ± 2.25	83.08^c^ ± 0.29	64.67^a^ ± 1.26	111.50^e^ ± 2.50	94.83^d^ ± 2.52	84.76^c^ ± 2.00	123.17^f^ ± 1.76	ND	ND
A420 (nm)	0.31^a^ ± 0	0.30^a^ ± 0	0.44^b^ ± 0.01	0.30^a^ ± 0	1.36^d^ ± 0	0.57^c^ ± 0	0.51^c^ ± 0	0.42^b^ ± 0	ND	ND
A520 (nm)	0.25^a^ ± 0	0.25^a^ ± 0	0.37^b^ ± 0.01	0.26^a^ ± 0	1.30^b^ ± 0	0.49^d^ ± 0	0.44^b^ ± 0	0.37^b^ ± 0	ND	ND
A620 (nm)	0.10^a^ ± 0	0.12^a^ ± 0	0.23^b^ ± 0.01	0.15^a^ ± 0	1.30^c^ ± 0	0.24^b^ ± 0	0.21^b^ ± 0	0.10^a^ ± 0	ND	ND
Tonality	1.29^c^ ± 0.11	1.22^b^ ± 0.03	1.19^b^ ± 0.03	1.17^b^ ± 0.06	1.05^a^ ± 0.02	1.16^b^ ± 0.04	1.17^b^ ± 0.04	1.14^b^ ± 0.05	ND	ND

*Values with different superscript roman letters (a–h) in the same row are significantly different according to the Tukey test (p < 0.05). ND. not determined*.

The two trials consisting in co-inoculating immobilized *S. cerevisiae* and free *O. oeni* (C3 sample) or free *S. cerevisiae* and free *O. oeni* (C5 sample) completed the AF and MLF after about 18 days post inoculation. In terms of time, these approaches produced an intermediate behavior between the fermentation evolution observed by all the samples employing immobilized *S. cerevisiae* and *O. oeni* cells (C1, C2, and C4) and the traditional sequential inoculation system using free cells of *S. cerevisiae* and *O. oeni* (C6) (Figures 2–4). Moreover, the use of sequential inocula of free *S. cerevisiae* cells and of *O. oeni* immobilized cells (C7 sample) allowed the end of AF and MLF fermentations after 34 days.

In all must fermentations performed using *S. cerevisiae* free cells, final yeast cell concentrations ranged between 7 × 10^7^ and 1.2 × 10^8^ CFU/mL (Figures 3D, 4A–C), whereas in samples inoculated with free *O. oeni* cells, bacterial concentrations ranged between 1.4 × 10^6^ and 2.5 × 10^7^ CFU/mL (Figures 3B,D, 4A).

### Fermentation parameters

The biochemical analysis of main compounds was carried out in the wines produced in all the above trials. Sugar consumption rate of C1 and C5 samples scored the highest value (23.4 and 26.44 g/L d, respectively), followed by C8 (21.15 g/L d), C2 (20.6 g/L d), C4 (13.75 g/L d), C6 (13.23 g/L d), C3 (11.74 g/L d), and C7 (7.06 g/L d) samples. C1 sample showed also the highest rate of glycerol production (0.52 g/L d), malic acid consumption (0.93 g/L d) and lactic acid production (0.58 g/L d) and volatile acidity production (0.06 g/L d; Table 2).

**Table 2:** **Fermentation metabolites production/consumption rates**.

	**C1**	**C2**	**C3**	**C4**	**C5**	**C6**	**C7**	**C8**	**C9**	**C10**
	**g/L Day**									
Malic acid consumption rate	0.93	0.93	0.19	0.13	0.24	0.16	0.1	0	0	0
Lactic acid production rate	0.58	0.54	0.1	0.06	0.12	0.1	0.08	0	0	0
Sugar consumption rate	23.4	20.6	11.74	13.75	26.44	13.23	7.06	21.15	0	0
Volatile acidity	0.06	0.05	0.03	0.02	0.03	0.02	0.015	0.03	0	0
Glycerol production rate	0.52	0.49	0.27	0.15	0.32	0.22	0.16	0.45	0	0

Ethanol content in fermented must samples obtained employing immobilized *S. cerevisiae* and *O. oeni*, in co-inoculum or sequential inoculums (C1, C2, and C5), ranged from 7.90 to 10.58 ± 0.02% (v/v), with a yield of about 43–53% considering an initial sugar content corresponding to 162.85 g/L (Table 1). Analogously, must samples inoculated with *S. cerevisiae* and *O. oeni* following the traditional sequential inoculation approach (C6) contained 11.32 ± 0.01% (v/v) of ethanol, which corresponded to a yield of 53% considering the initial sugar content of 214.34 g/L (C10). The separate microbial immobilization of *S. cerevisiae* and *O. oeni* in alginate beads and their use in co-inoculum or in sequential inoculum approaches did not affect volatile acidity. In fact, very low levels of volatile acidity were produced in all fermented must samples (Table 1). All fermentations produced wines with pH values (3.23–3.47) and tartaric and citric acid concentrations were slightly different among samples. In particular, lactic acid was produced in a detectable quantity in all samples containing *O. oeni*, but not in C8 sample inoculated by only *S. cerevisiae*, thus indicating that it derived principally by bacterial malic acid decarboxylation. A corresponding high level of ethyl lactate was measured in samples inoculated with *O. oeni* immobilized in alginate beads (C1, C2, C4, and C7). Even low levels of glycerol ranging from 4.15 to 6.36 g/L were produced in all samples, as expected, this compound represented the second major product of AF. Moreover, a reduction of 15–20% of wine color was observed (Absorbance at 420, 520, 620 nm, and tonality).

### Volatile analyses

Quantitative data of the volatile compounds found in wines are shown in Tables 3A,B. These tables also show the perception thresholds of volatiles and their corresponding odor descriptors. Thirty-nine volatile components were identified and quantified in the analyzed wines. The main classes are alcohols, volatile fatty acids, esters, while aldehydes, terpens, sulfur compound, lactones, volatile phenols, and pyrazine were present in low concentrations.

**Table 3A:** **Quantification of volatile compounds identified in C1-C5 wines**.

**Compounds**	**OTH mg/L**	**Odor description**	**References**	**C1 mg/L ± sd**	**C2 mg/L ± sd**	**C3 mg/L ± sd**	**C4 mg/L ± sd**	**C5 mg/L ± sd**
**ALCOHOLS**								
1-propanol	9	Pungent, harsh			1.47ab ± 0.44	1.53b ± 0.17	1.11ab ± 0.22	0.87ab ± 0.40
			Penaido et al., 2006	2.21b ± 0.80				
2-methyl-1-propanol	40	Fusel, spiritous	Gómez-Míguez et al., 2007	7.72b ± 1.70	5.60b ± 0.43	8.70b ± 1.11	7.18b ± 1.45	6.07b ± 2.85
1-butanol	150	Fusel, spiritous	Penaido et al., 2006	0.52a ± 0.26	0.28a ± 0.11	0.67b ± 0.26	0.45b ± 0.36	0.35b ± 0.12
Isoamylic alcohols	30	Harsh, nail polish	González Álvarez et al., 2011	85.66b ± 14.13	73.55b ± 7.22	99.05b ± 8.66	84.76b ± 6.54	75.79b ± 8.98
Hexanol	4	Herbaceous	Rocha et al., 2004	0.32b ± 0.10	0.28b ± 0.03	0.15ab ± 0.04	0.30b ± 0.07	0.10b ± 0.04
Heptanol	2.5	Oily		0.26b ± 0.05	0.21b ± 0.03	0.43a ± 0.18	0.45a ± 0.18	0.44a ± 0.03
(D,L)- butan-2,3-diol	150	Fruity	Penaido et al., 2006	4.63b ± 2.15	2.65ab ± 0.75	3.89ab ± 3.30	2.33ab ± 0.40	1.16ab ± 0.65
(R,S)- butan-2,3-diol	150	Fruity	Penaido et al., 2006	1.79a ± 0.22	0.89a ± 0.52	0.94a ± 0.23	0.64a ± 0.33	0.54a ± 0.20
Benzylalcohol	200	Flowery, sweet	González Álvarez et al., 2011	ND	1.39b ± 0.06	0.81a ± 0.24	1.57b ± 0.26	ND
Phenyl ethyl alcohol	10	Rose	Rocha et al., 2004	49.33c ± 9.41	38.64bc ± 6.60	52.31d ± 4.27	46.89c ± 5.90	49.13c ± 6.15
Total amounts				152.44 ± 28.82	124.96 ± 16.20	168.48 ± 18.46	145.68 ± 15.71	134.45 ± 19.42
**FATTY ACIDS**								
Acetic acid	200	Vinegar	Guth, 1997	9.56d ± 5.60	5.44c ± 0.84	14.57e ± 5.46	10.11d ± 3.52	3.31b ± 0.19
2-methyl propanoic acid	2.3	Fatty, rancid	Gómez García-Carpintero et al., 2011b	1.23b ± 0.36	0.92ab ± 0.12	1.62ab ± 0.17	1.13ab ± 0.29	1.07ab ± 0.51
Butanoic acid	2.2	Fatty-rancid, sweaty	Rocha et al., 2004	1.11b ± 0.18	0.90ab ± 0.12	1.39c ± 0.47	0.89ab ± 0.25	0.71ab ± 0.33
3-methyl butanoic acid	1.5	Fatty-rancid, cheesy	Moyano et al., 2002	3.13b ± 0.87	1.95ab ± 0.15	2.87b ± 0.78	2.37ab ± 0.71	2.40ab ± 0.63
Hexanoic acid	8	Rancid, grass, fruity	Rocha et al., 2004	10.79e ± 2.59	10.72e ± 1.30	13.09f ± 2.71	9.34d ± 1.32	8.64c ± 1.58
Octanoic acid	0.5	Fatty acid, dry, dairy	Rocha et al., 2004	14.98bc ± 5.42	15.02bc ± 3.56	16.79c ± 4.07	11.98bc ± 2.34	10.81bc ± 3.40
Decanoic acid	1.4	Fatty acid, dry, woody	Rocha et al., 2004	3.29a ± 0.46	3.01a ± 0.18	2.19a ± 0.22	2.88a ± 1.08	4.52a ± 0.75
Benzoic acid	1	Chemical		6.54ab ± 2.12	5.18ab ± 0.27	6.95b ± 0.64	5.22ab ± 0.41	3.59ab ± 0.46
Total amounts				50.62 ± 17.61	43.14 ± 5.71	59.46 ± 14.53	43.92 ± 9.91	35.06 ± 7.83
**ESTERS**								
Ethyl-butanoate	0.02	Floral, fruity	Guth, 1997	1.31b ± 0.54	1.07ab ± 0.03	1.20b ± 0.20	0.81ab ± 0.10	0.99ab ± 0.31
Isoamyl acetate	0.03	Banana, pear	Günata et al., 1985	4.33b ± 1.65	4.33b ± 0.32	5.07b ± 0.93	2.15ab ± 0.24	5.22b ± 0.74
Ethyl hexanoate	0.014	Green apple	Günata et al., 1985	1.70ab ± 0.43	1.30ab ± 0.07	1.95b ± 0.29	1.19ab ± 0.41	2.04b ± 0.21
Ethyl lactate	154.6	Lactic	Gómez-Míguez et al., 2007	6.03cd ± 1.16	5.89cd ± 0.05	4.41c± 0.54	6.68d ± 0.93	2.29b ± 0.48
Ethyl octanoate	0.6	Sweet soap	Rocha et al., 2004	2.22b ± 0.87	3.35b ± 0.81	6.22c ± 0.20	3.10b ± 0.10	6.71c ± 0.64
3-hydroxy ethyl butanoate	20	Fruity	González Álvarez et al., 2011	0.61b ± 0.26	0.35ab ± 0.07	0.69b ± 0.20	0.33ab ± 0.07	0.39ab ± 0.12
Ethyl decanoate	0.2	Fruity		1.17ab ± 0.32	1.37ab ± 0.37	0.36ab ± 0.10	0.85ab ± 0.29	5.78b ± 2.26
Diethyl succinate	200	Fruity, melon	González Álvarez et al., 2011	0.46ab ± 0.23	0.38ab ± 0.05	0.54b ± 0.04	0.45b ± 0.08	1.30c ± 0.15
Ethyl-9-decenoate	ND			0.48ab ± 0.08	0.33ab ± 0.07	1.05ab ± 0.40	1.10ab ± 0.36	2.45b ± 0.81
Phenyl acetate	0.25	Flowery, rose, fruity	Guth, 1997	0.94abc ± 0.34	0.86abc ± 0.05	1.30bc ± 0.43	0.58abc ± 0.20	1.41c ± 0.12
Monoethyl succinate	ND	Chocolate		5.71abc ± 0.08	2.93ab ± 0.38	7.11bc ± 0.40	9.28bc ± 2.45	5.25abc ± 1.60
Total amounts				24.42 ± 5.97	22.17 ± 2.27	29.92 ± 3.73	26.48 ± 5.23	33.81 ± 7.44
**ALDEHYDES/KETONS**								
Acetoin	150		Moyano et al., 2002	0.49a ± 0.24	ND	1.01ab ± 0.10	0.47a ± 0.13	0.95ab ± 0.26
Furfural	3	Caramel		0.34 ± 0.08	0.23 ± 0.03	0.29 ± 0.12	0.23 ± 0.07	0.31 ± 0.07
Benzaldehyde	2	Bitter almond	Gómez García-Carpintero et al., 2011b	0.73a ± 0.25	0.63a ± 0.06	0.75a ± 0.24	0.49a ± 0.20	0.35a ± 0.06
Methyl-2-furoate	ND			0.87a ± 0.39	0.60a ± 0.09	0.81a ± 0.18	0.42a ± 0.13	0.38a ± 0.54
Total amounts				2.43 ± 0.96	1.46 ± 0.17	2.86 ± 0.64	1.61 ± 0.53	1.99 ± 0.93
**LACTONES**								
Butyrolactone	0.035	Sweet, toast, caramel	Moyano et al., 2002	0.53a ± 0.24	ND	1.78a ± 0.044	2.79a ± 0.61	ND
**SULFUR COMPOUND**								
3-methylthio-1-propanol	2	Potato, baked cabbage	Guth, 1997	1.55ab ± 0.70	0.71ab ± 0.11	2.32b ± 0.70	1.99ab ± 0.50	1.36ab ± 0.98
**PHENOLS**								
4-vinyl guaiacol	0.38-1.1	Black pepper, species	Rocha et al., 2004	3.68b ± 0.72	3.41ab ± 0.92	3.53ab ± 1.51	0.68ab ± 0.28	1.72ab ± 0.46
**PYRAZINE**								
2-methoxy-6-methyl pyrazine								
**TERPENS**								
2,6 dimethyl-7-octene 2,6 diol	0.250		Vilanova and Sieiro, 2006	0.64a ± 0.25	0.59a ± 0.06	0.86a ± 0.40	ND	0.30a ± 0.42
4H-piran-4-one-2,3 dihydro-3,5 dihydroxy	ND			2.50a ± 0.92	1.76a ± 0.22	3.55a ± 1.32	1.32a ± 0.45	1.20a ± 0.48
Total amounts				3.14 ± 1.17	2.35 ± 0.28	4.41 ± 1.72	1.32 ± 0.45	1.49 ± 0.90

*Different letters in the same row indicate statistical differences (p < 0.05) using the Tukey test; ND, not determined; Data are average of three replicates ± standard deviation (SD); OTH, odor threshold*.

**Table 3B:** **Quantification of volatile compounds identified in C6-C10 wines**.

**Compounds**	**OTH mg/L**	**Odor description**	**Ref**.	**C6 mg/L ± sd**	**C7 mg/L ± sd**	**C8 mg/L ± sd**	**C9 mg/L ± sd**	**C10 mg/L ± sd**
**ALCOHOLS**								
1-propanol	9	Pungent, harsh	Penaido et al., 2006	1.13a ± 0.53	0.99a ± 0.17	1.19a ± 0.17	Nd	Nd
2-methyl-1-propanol	40	Fusel, spiritous	Gómez-Míguez et al., 2007	7.74a ± 1.60	8.01a ± 1.02	8.34a ± 0.24	Nd	Nd
1-butanol	150	Fusel, spiritous	Penaido et al., 2006	0.43a ± 0.10	0.08a ± 0.11	0.39a ± 0.10	Nd	Nd
Isoamylic alcohols	30	Harsh, nail polish	González Álvarez et al., 2011	97.45b ± 7.80	93.52b ± 5.04	88.00b ± 9.71	34.11a ± 3.70	37.05a ± 4.11
Hexanol	4	Herbaceous	Rocha et al., 2004	0.11a ± 0.02	0.27b ± 0.04	Nd	Nd	Nd
Heptanol	2.5	Oily		0.42a ± 0.03	0.39a ± 0.03	0.46a ± 0.05	0.29a ± 0.08	0.24a ± 0.04
(D,L)- butan-2,3-diol	150	Fruity	Penaido et al., 2006	1.56a ± 1.03	1.40a ± 1.03	1.28a ± 0.21	Nd	Nd
(R,S)- butan-2,3-diol	150	Fruity	Penaido et al., 2006	0.96a ± 0.41	0.85a ± 0.11	0.54a ± 0.04	Nd	Nd
Benzylalcohol	200	Flowery, sweet	González Álvarez et al., 2011	0.43a ± 0.61	Nd	Nd	0.72a ± 0.25	0.98a ± 0.20
Phenyl ethyl alcohol	10	Rose	Rocha et al., 2004	56.56d ± 4.49	22.59b ± 3.10	49.48c ± 4.60	4.35a ± 1.12	5.66a ± 0.11
Total amounts				166.79 ± 16.62	128.1 ± 10.65	149.68 ± 15.12	39.47 ± 5.15	43.93 ± 4.46
**FATTY ACIDS**								
Acetic acid	200	Vinegar	Guth, 1997	6.21c ± 2.22	7.34c ± 1.15	4.52c ± 1.71	1.64a ± 0.70	Nd
2-methyl propanoic acid	2.3	fatty, rancid	Gómez García-Carpintero et al., 2011b	1.09ab ± 0.22	1.08ab ± 0.07	0.95ab ± 0.21	0.56a ± 0.13	0.70a ± 0.05
Butanoic acid	2.2	Fatty-rancid, sweaty	Rocha et al., 2004	0.84b ± 0.13	0.69b ± 0.06	0.83b ± 0.22	Nd	0.31a ± 0.06
3-methyl butanoic acid	1.5	Fatty-rancid, cheesy	Moyano et al., 2002	2.74b ± 0.35	2.61ab ± 0.07	2.47ab ± 0.23	0.45a ± 0.10	0.49a ± 0.06
Hexanoic acid	8	Rancid, grass, fruity	Rocha et al., 2004	10.35e ± 3.23	6.16b ± 0.57	9.53d ± 3.30	0.47a ± 0.10	0.77a ± 0.11
Octanoic acid	0.5	Fatty acid, dry, dairy	Rocha et al., 2004	14.12ab ± 4.82	8.59a ± 1.33	11.87ab ± 2.60	Nd	Nd
Decanoic acid	1.4	Fatty acid, dry, woody	Rocha et al., 2004	2.51a ± 0.66	4.59a ± 0.88	2.25a ± 0.26	Nd	Nd
Benzoic acid	1	Chemical		5.06ab ± 0.21	4.49ab ± 0.91	Nd	1.77ab ± 0.45	2.71ab ± 0.15
Total amounts				42.91 ± 9.84	35.54 ± 5.04	32.42 ± 8.53	4.89 ± 1.48	4.99 ± 0.43
**ESTERS**								
Ethyl-butanoate	0.02	Floral, fruity	Guth, 1997	1.04ab ± 0.35	0.69a ± 0.13	1.19b ± 0.35	Nd	Nd
Isoamyl acetate	0.03	Banana, pear	Günata et al., 1985	3.74ab ± 0.11	1.65a ± 0.24	3.99b ± 0.16	Nd	Nd
Ethyl hexanoate	0.014	Green apple	Günata et al., 1985	1.24b ± 0.50	0.55a ± 0.05	1.19b ± 0.30	Nd	Nd
Ethyl lactate	154.6	Lactic	Gómez-Míguez et al., 2007	4.65b ± 0.42	6.06b ± 0.27	0.60a ± 0.08	Nd	Nd
Ethyl octanoate	0.6	Sweet soap	Rocha et al., 2004	2.06a ± 0.34	1.33a ± 0.27	2.41a ± 0.80	Nd	2.31a ± 0.05
3-hydroxy ethyl butanoate	20	Fruity	González Álvarez et al., 2011	0.39a ± 0.05	0.36a ± 0.10	0.48a ± 0.06	Nd	Nd
Ethyl decanoate	0.2	Fruity		0.80a ± 0.04	1.03a ± 0.06	1.03a ± 0.04	Nd	Nd
Diethyl succinate	200	Fruity, melon	González Álvarez et al., 2011	0.47b ± 0.15	0.39a ± 0.07	0.38a ± 0.04	Nd	Nd
Ethyl-9-decenoate	Nd			0.92a ± 0.12	0.84a ± 0.08	1.17a ± 0.10	Nd	Nd
Phenyl acetate	0.25	Flowery, rose, fruity	Guth, 1997	0.82a ± 0.33	0.41a ± 0.05	0.72a ± 0.08	Nd	Nd
Monoethyl succinate	Nd	Chocolate		7.23b ± 0.32	10.20c ± 1.45	4.18a ± 0.56	Nd	Nd
Total amounts				23.38 ± 2.73	23.51 ± 2.77	17.35 ± 2.57		2.31 ± 0.05
**ALDEHYDES/KETONS**								
Acetoin	150		Moyano et al., 2002	1.36b ± 0.23	0.89a ± 0.16	Nd	1.09a ± 0.05	1.17a ± 0.06
Furfural	3	Caramel		0.37a ± 0.07	0.47a ± 0.05	0.29a ± 0.05	1.56a ± 0.07	1.84a ± 0.04
Benzaldehyde	2	Bitter almond	Gómez García-Carpintero et al., 2011b	0.36a ± 0.08	0.34a ± 0.05	0.55a ± 0.05	0.47a ± 0.07	0.48a ± 0.05
Methyl-2-furoate	ND			0.93a ± 0.16	0.73a ± 0.14	0.60a ± 0.07	Nd	Nd
Total amounts				3.01 ± 0.54	2.43 ± 0.40	1.44 ± 0.17	3.13 ± 0.19	3.48 ± 0.15
**LACTONES**								
Butyrolactone	0.035	Sweet, toast, caramel	Moyano et al., 2002	0.45a ± 0.07	Nd	0.52a ± 0.05	1.07a ± 0.06	1.16a ± 0.07
**SULFUR COMPOUND**								
3-methylthio-1-propanol	2	Potato, baked cabbage	Guth, 1997	2.06ab ± 0.05	1.94ab ± 0.05	1.72ab ± 0.05	0.27a ± 0.04	0.32a ± 0.08
**PHENOLS**								
4-vinyl guaiacol	0.38-1.1	Black pepper, species	Rocha et al., 2004	Nd	Nd	0.96 a	Nd	Nd
**PYRAZINE**								
2-methoxy-6-methyl pyrazine							4.80b ± 0.12	1.20a ± 0.05
**TERPENS**								
2,6 dimethyl-7-octene 2,6 diol	0.250		Vilanova and Sieiro, 2006	0.56a ± 0.05	0.53a ± 0.07	0.50a ± 0.04	0.47a ± 0.07	0.59a ± 0.10
4H-piran-4-one-2,3 dihydro-3,5 dihydroxy	ND			1.87a ± 0.15	1.24a ± 0.16	3.34a ± 0.12	1.64a ± 0.06	2.58a ± 0.06
Total amounts				2.44 ± 0.20	1.77 ± 0.23	3.84±	2.11 ± 0.13	3.16 ± 0.16

*Different letters in the same row indicate statistical differences (p < 0.05) using the Tukey test; ND, not determined; Data are average of three replicates ± standard deviation (SD); OTH, odor threshold*.

Well-known by-products of yeast metabolism were the most abundant substances. In fact, alcohols are quantitatively the largest group of volatile compounds and they were present in a higher amounts in C1 (152.44 mg/L), C3 (168.48 mg/L), and C6 (166.79 mg/L) samples, without any statistically significant difference among these samples. The highest concentrations were observed for isoamylic alcohols (73.55–99.05 mg/L), phenyl ethyl alcohol (4.35–56.56 mg/L), and 2-methyl-1-propanol (5.60–8.70 mg/L). Phenyl ethyl alcohol, key compound in the floral flavors of wines, was the second most abundant alcohol in C6 (56.56 mg/L), C3 (52.31 mg/L), and C1 (49.34 mg/L). In all fermented samples, isoamyl-alcohols and phenyl ethyl alcohol, the most important volatile compounds, were present in concentrations exceeding the odor threshold, producing positive impact in wine aroma (Tables 3A,B).

Eight different volatile fatty acids were identified and C1 (total amount 50.62 mg/L) and C3 (total amount 59.46 mg/L) samples showed the highest concentrations of these compounds. Statistically significant differences have been observed among the samples for all acids, except for decanoic acid. Acetic acid was the most abundant acid (1.64–14.57 mg/L), being present at levels lower than its perception threshold (200 mg/L), next followed by octanoic acid (8.59–16.79 mg/L) and hexanoic acid (0.47–13.09 mg/L). Ethyl esters of fatty acids and acetates were the second abundant group of volatile compounds in wines with 11 different identified components. Most of them are ethyl esters of fatty acids produced during the AF and the concentrations of many of them were significant different (*p* < 0.05), among wine samples. Since all of them (ethyl butanoate, hexanoate, octanoate, and decanoate) surpassed the detection threshold in all wines, except for ethyl lactate and 3-hydroxy ethyl butanoate, consequently, they are expected to have a great influence on the aroma of tested wines (Tables 3A,B). Isoamyl acetate and phenyl acetate (originating by the reaction of the acetyl-CoA with higher alcohols) showed high concentration levels, exceeding their odor threshold in all samples, whereas aldehydes remained quantitatively very limited (Tables 3A,B).

Among fatty acids, also produced during fermentation, butanoic, 3-methyl butanoic, hexanoic, octanoic, decanoic, and benzoic acids were quantified in concentrations exceeding the odor threshold and contributing with fruity, fatty, rancid, and cheese notes on wine odor profile. Also among terpenes, strongly influencing the varietal aroma, 2,6 dimethyl-7-octene 2,6 diol was identified in all wines with an OAV > 1. Since low levels of acetoin and 2,3 butanediol were detected in all wine samples, these compounds did not affect aroma with the unpleasant “*buttery*” attribute.

Volatile phenol (4-vinyl guaiacol) was detected with an OAV > 1 in all wine samples except in C6 and C7 wines. This compound can be responsible of spicy aromatic notes.

### Principal component analysis

PCA was carried out on the 10 samples using the principal fermentation parameters reported in Tables 1, 2, in order to produce a multivariate analysis of the evolution of chemical compounds and the fermentation rates linked to production/consumption of main fermentation metabolites.

**Figure 5:**
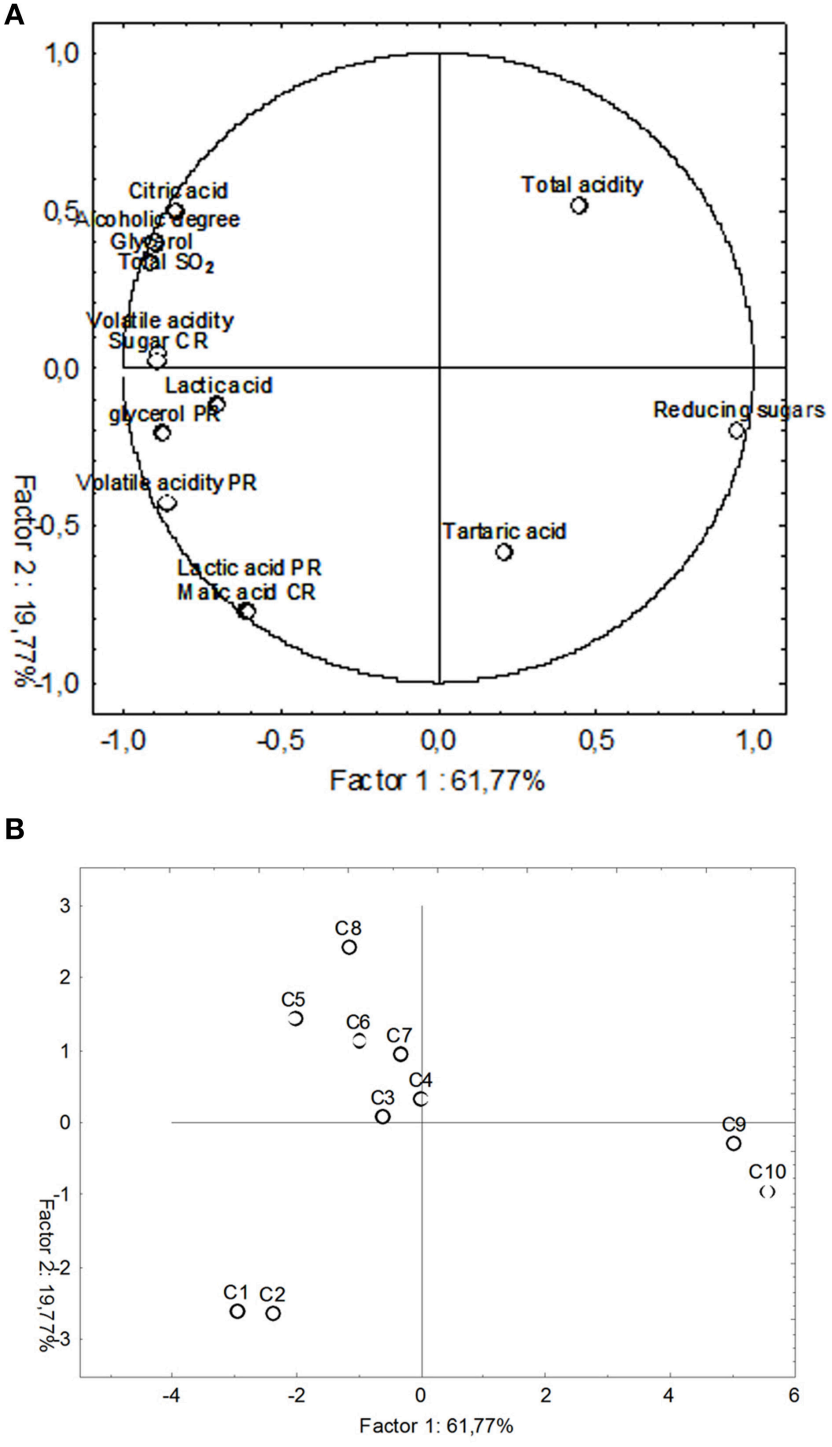
**PCA of fermentation parameters associated with all fermentation samples**. PCA variables were the data obtained from the analysis of concentration and production rates (PR) and consumption rates (CR) of chemical compounds at the end of fermentation. The figure displays the sample scores and variable loadings in the planes formed by PC1–PC2. **(A)** Projection of the variables; **(B)** Projection of the cases.

In Figure 5 bi-plots displaying PC1 vs. PC2 indicated that the samples considered in this study were grouped into three main clusters: the cluster 1 (C1 and C2) and 2 (C3, C4, C5, C6, C7, and C8) were divided by the PC2 and were localized in quadrants 3 and 4, respectively; the cluster 3 (C9 and C10 samples), separated from the two others group by PC1 localized in quadrant 2.

**Figure 6:**
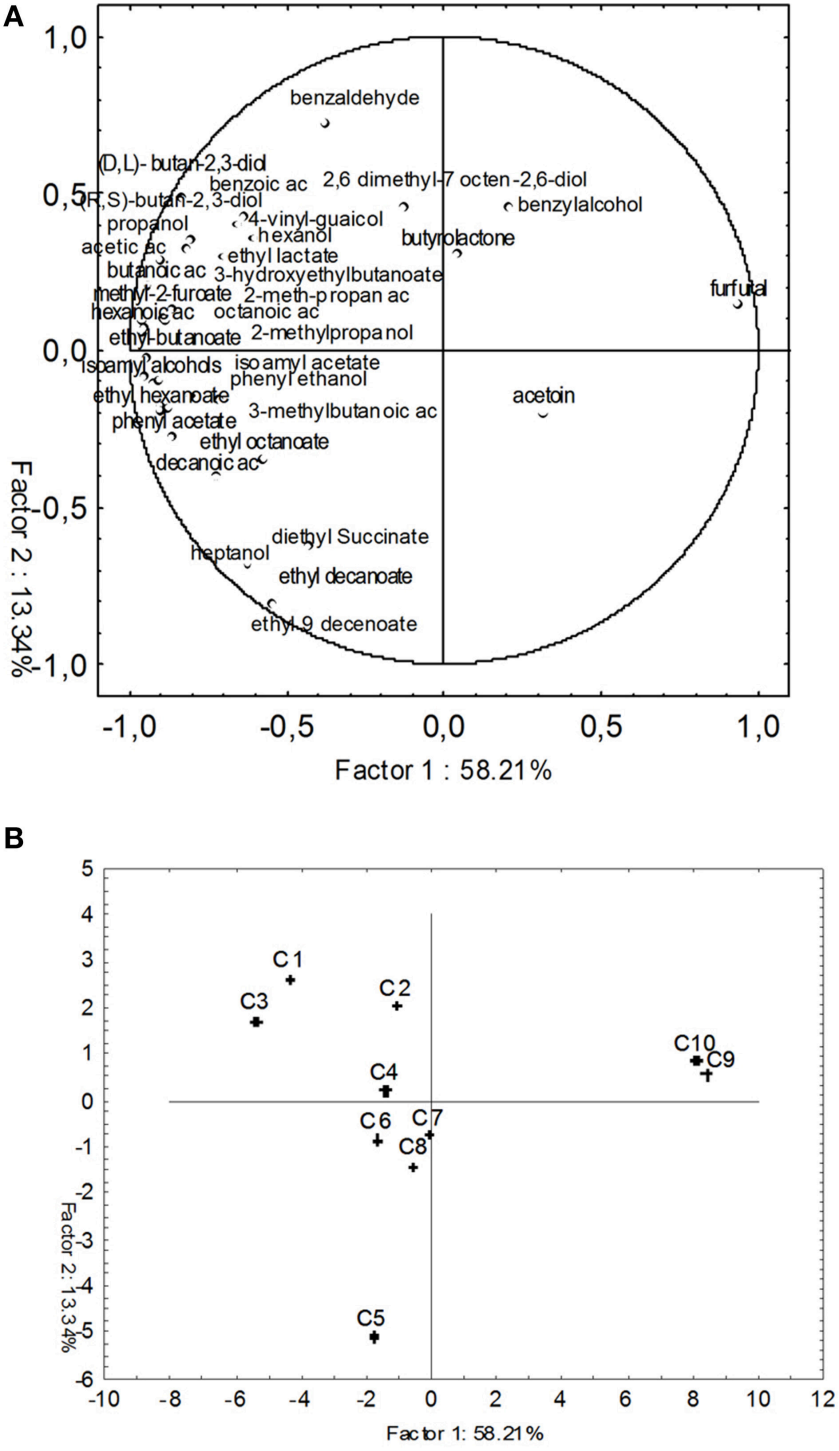
**PCA of volatile compounds associated with all tested fermentation samples**. Score plot of variables (concentration of volatile molecules) and the end of fermentation in the plane formed by the first two principal components (PC1 against PC2). **(A)** Projection of the variables; **(B)** Projection of the cases.

In order to correlate volatiles data with the different inoculum strategies used in this work, a PCA analysis was performed on the complete SPME/GC-MS data matrix of each wine sample (Figure 6). PC1 discriminated wine samples (C1–C8) which lied on the negative semi-axis of the first component form the two must controls (C9 and C10) for the high content of volatile compounds. The second dimension allowed to separate two clusters A and B: group A (C5, C6, C7, and C8) on the negative semi-axis of PC2 and group B (C1, C2, C3, and C4) on the positive semi-axis of PC2. Group A in the third quadrant along negative PC2 was characterized by the presence of esters, whereas, group B, localized in the fourth quadrant is mainly characterized by highly flavoring compounds such as acids, esters, terpens, alcohols, lactones, and aldehydes.

### Radar plot

**Figure 7:**
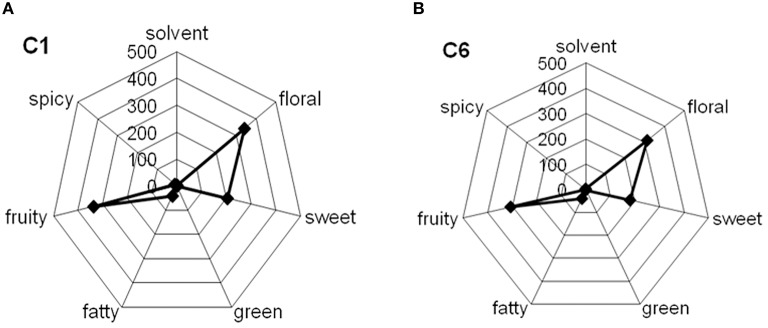
**Odor chemical profile of wines calculated by adding the odor descriptor of the total compounds concentration grouped in chemical class**. Radar plot of the sensory descriptors associated to total concentrations of all classes of volatiles associated to Negroamaro wine obtained after **(A)** inoculation with *Oenococcus oeni* strain Lalvin VP41™ and *Saccharomyces cerevisiae* strain Lalvin ICV-D254® co-immobilized in alginate beads (sample C1), **(B)** inoculation of free (10^6^ CFU/ml) *S. cerevisiae* cells, followed, at the end of AF, by free cells (10^6^ CFU/ml) of *O. oeni* (sample C6).

Odorous compounds detected in all analyzed wine and must samples with similar sensory descriptors were grouped into classes, denoted aromatic series (Tables 3A,B). In these Tables, one or more membership sensorial classes was affiliated to each compound. In this respect, solvent, floral, sweet, green, fatty, fruity, and spicy odor series were chosen for the description of wines aroma. Figure 7 reports the radar plot representation of the odor series associated to C1 and C6 wine samples.

C1 wine sample, produced by yeast and bacteria co-immobilization strategy, showed a sensorial profile comparable with that produced by sequential inoculum strategy (C6 sample). In particular, C1 sample showed also spicy (phenols, 4 vinyl guaiacol) notes that are completely absent in C6 wine (Tables 3A,B).

## Discussion

In this study, for the first time the co-immobilization strategy in alginate beads of two commercial strains of *S. cerevisiae* and *O. oeni* was applied for the production of red wine. Although the parameters adopted for yeasts immobilization (initial cell biomass, CaCl_2_, and the sodium alginate concentrations) were than those described by Bleve et al. (2008, 2011), the immobilization conditions for *O. oeni* were instead optimized. The best co-immobilization conditions for *S. cerevisiae* and *O. oeni* cells corresponded to an inoculum equivalent to 10^6^ CFU/mL of each *O. oeni* and of *S. cerevisiae* in 3% (w/v) calcium alginate. The data reported in this study showed that, after immobilization, yeast and bacterial metabolic activities were enhanced in comparison with those inoculated in free cells form. In fact, the immobilization enhances biological stability, the tolerance to external stress conditions, the resistance to by-products deriving from cell metabolism and that (this can result toxic to the same cells; Bleve et al., 2008, 2011). This observation was particularly important for *O*. *oeni* since this microorganism is exposed to very difficult constraints (like pH, ethanol, SO_2_, medium chain fatty acids, nutrient depletion, etc.) that can negatively affect its growth and metabolic activities and, consequently, the occurrence of MLF in wines (Alexandre et al., 2004). After comparison with the different supports (Kourkoutas et al., 2004), calcium alginate was chosen as material to encapsulate yeast and bacteria cells, since it is cheap and of food grade purity. This strategy offers the possibility to encapsulate cells mimicking the environment of large flocs, producing a high mass:surface ratio, protecting the inside aggregated cells from stress by an outer layer of sacrificial cells (Sun et al., 2007).

This effect cannot be obtained by the use of carrier materials (glass beads, wood chips, etc.) where cells adher to the matrix surface, leaving them to be directly exposed to the external stresses (Nedović et al., 2015).

Several examples of yeast immobilization in alginate beads have been proposed to obtain a suitable biocatalyst for mead production by diluted honey (Pereira et al., 2014), for making of pomegranate (Sevda and Rodrigues, 2011), and cagaita-derived (Oliveira et al., 2011) wines, for production of cabernet sauvignon and pinot noir young wines (Andrade Neves et al., 2014).

The high AF and MLF fermentation rates maintained in all the three different wine production cycles using co-immobilized *S. cerevisiae* and *O. oeni* in this study indicated that co-immobilization of yeast and bacterial cells did not induce alterations in physiology and metabolic activity and in cell growth. These evidences demonstrated that a good balance exists among fermentation rates, main fermentation metabolites in the final product, and suitable internal mass transfer in co-immobilization of yeasts and bacteria in calcium alginate, as suggested by Scott and O'Reilly (1996).

In a previous paper, Servetas et al. (2013) used tubular delignified cellulosic material and wheat starch gel to respectively entrap, in two overlapped layers, *O. oeni* and *S. cerevisiae* cells, which were able to carry out simultaneous AF and MLF at low temperature (10°C), without problems deriving from the biological competition in the same niche.

Co-immobilization strategy can be useful to significantly reduce the time necessary to obtain a complete AF and MLF. These results confirmed that when yeast cells are immobilized they showed a faster consume of glucose than free cells, due to a stable pattern of gene expression characterized by higher expression of genes involved in glycolysis, stress resistance and cell wall remodeling than planktonic cells (Nagarajan et al., 2014). In addition, Parascandola et al. (1997) and Junter et al. (2002) reported that immobilization produces significant changes in the cell proteome and gene expression that have relevant impact on cell wall and cytoplasmic membrane composition and architecture, finally producing deep impact on cell stress resistance.

According to what observed by Rodriguez-Nogales et al. (2012), where MLF was enhanced by the immobilization of *O. oeni* in Lentikats, the proposed approach in this paper can open interesting perspectives, since it speed up the wine production by shortening the time needed for MLF completion.

Statistical analyses used in this paper demonstrated that C1 and C2 wine samples were strongly distinguishable from all the other wines when metabolites production and/or their consumption rates were considered (Figure 5).

Although food grade alginate beads immersed in grape must can dilute metabolites and color of must and wines, as previously reported by Genisheva et al. (2013), the proposed approach produced final products with sensorial characteristics, determined by OAVs, not different from the wine obtained using the traditional sequential inoculum procedure.

The perception thresholds and descriptors for each aroma compound studied as previously reported (Brugirard et al., 1991; Guth, 1997; López et al., 1999; Kotseridis and Baumes, 2000; Ferreira et al., 2001). Each compound was assigned to one or several aroma series, depending on its principal odor descriptors; the solvent, floral, sweet, green (vegetal or herbaceous), fatty, fruity, and species were chosen for this purpose on account of their extensive use for describing and distinguishing red wines in terms of aroma by specialized journals and tasters (Mijares, 1987; Peynaud, 1987; Peris and Masats, 2000).

However, the projection of the cases onto the first two axes of PCA, performed on volatiles, showed that wines produced using beads as support for yeasts and bacteria immobilization (C1, C2, C3, and C4) were more complex in terms volatile compounds concentrations (acids, esters, terpens, alcohols, lactones, and aldehydes) than wines obtained using free cells inocula (C5, C6, C7, and C8), mainly characterized by esters (Figure 6).

Higher alcohols, mainly formed during AF, are the largest group of aroma compounds, contributing, especially by the synergistic effect of the matrix (Verstrepen et al., 2003b) with fruity characters, when they are in optimal levels (< 300 mg/L). They are also at the basis of volatile ester formation (Verstrepen et al., 2003a). These compounds did not exceed in any tested wines the threshold of 400 mg/L, avoiding to produce strong and pungent smell and taste and herbaceous notes (Ribereau-Gayon et al., 2000). Isoamyl alcohol may contribute to the complexity of aroma wine, although at very high levels, it can produce unpleasant notes. Among the aliphatic alcohols, 3-methyl-1-butanol showed the highest concentration in all studied wines; 2-Phenylethanol is formed principally by yeast metabolism (Etievant, 1991), has a floral aroma with roses notes. In all studied wines this compound exceeded its olfactory threshold (10 mg/L, Guth, 1997).

Although acetaldehyde is the most important volatile aldehyde for flavor in wines (Lambrechts and Pretorius, 2000), it resulted not detectable in all tested wines, probably because, after its production during the active yeast growth phase, it was sequestrated by the same yeast cells and used to furtherly produce ethanol (Verstrepen et al., 2003a).

The ethylic esters of the fatty acids (ethyl butanoate, hexanoate, octanoate, and decanoate) and the acetates of the higher alcohols (isoamyl acetate and phenyl acetate) are two groups of compounds of undoubted importance in the wine aroma, as their nuances coincide with the fruity, perfume-like, and candy descriptors of the wines. These compounds are important in young wine aroma and are among key compounds in the fruity flavors of wines (Rapp and Mandery, 1986). The presence of other esters, specifically ethyl acetate, phenylacetate, although exhibiting OAVs lower than one, also could contribute to the fruity character of analyzed wines. In fact, as already reported by Genisheva et al. (2014), immobilization system contribute to enhance the concentrations of isoamyl acetate, the ethyl esters ethyl hexanoate, ethyl octanoate, and ethyl decanoate in the final products above their perception thresholds, conferring to wines sweet and fruity flavors.

All volatile fatty acids detected were present at concentrations above 50 mg/L. Fatty acids have been described as giving rise to fruity, cheesy, fatty and rancid notes. Although, C6–C10 fatty acids are usually related to the appearance of negative odors, they are very important for aromatic equilibrium in wines because they oppose the hydrolysis of the corresponding esters (Torrens et al., 2008), and their presence plays an important role in the complexity of the aroma (Shinohara, 1985). Both esters and acetates have a key importance in the whole wine aroma impressing a characteristic fruity notes (Rapp and Versini, 1991; Swiegers and Pretorius, 2005).

The 4-vinyl-guaiacol was detected in all wines, with the exception of C6, C7, C9, and C10 samples. In white wines and at high concentrations, vinylphenols can be responsible for heavy “pharmaceutical” odors (Chatonnet et al., 1993), but at low and moderate concentration they can be related with pleasant spicy aroma. In this sense Grando et al. (1993), found that 4-vinil-guayacol was the main responsible for the spicy aroma of Gewurztraminer's wines.

Similar results were already obtained using yeast cells entrapped in sodium alginate and k-carrageenan for the production of rosé sparkling wine that resulted similar in sensory characteristics to the traditional products, but produced in reduced time (Tataridis et al., 2005).

In the presented screening of different inocula strategies of yeasts and bacteria for wine-making, aroma produced by free or immobilized cells in wine has been evaluated by gas chromatography: these chemical analyses of volatile compounds are suitable to produce important information about the compounds with odor-active potential. However, in agreement with the suggestions of Nedović et al. (2015), the actual sensory traits of wines produced by co-immobilization strategy will be evaluated employing trained panel and consumers, in order to obtain acceptable products to be proposed on the market.

Experiments are now under the way to set up a simple procedure to dry the co-immobilization system, in order to reduce the very limiting dilution effect on must metabolites, ensuring the maintenance of yeasts and bacteria viability and fermentation efficiency.

## Conclusions

Co-immobilization of *S. cerevisiae* and *O. oeni* allowed to perform a efficient fermentation process, eliminating non-productive cell growth phase, producing a biocatalyst that can be reused several times, sensitively reducing the time of the process, opening in the future the possibility to develop continuous process. Co-immobilization strategy produced a wine with organoleptic profiles comparable with that produced with the co-inoculation and the sequential inoculation strategies in free form.

Co-immobilization of *S. bayanus* and *Leuconostoc oenos* in Ca- alginate matrix, has already been used to optimize a continuous fermentation process for cider production (Nedovic et al., 2000). Genisheva et al. (2014) developed a continuous process consisting in sequential AF and MLF by the implementation of distinct packed-bed reactors containing immobilized *S. cerevisiae* on grape stems/skins, and *O. oeni* on grape skins, respectively.

This study individuates the most promising strategy to immobilize yeasts and *O. oeni* in a lab micro-vinification scale. The future step will be to test the suitability of co-immobilization strategy (in alginate or other matrices) to produce wines in pilot-scale, that can be more representative of actual conditions occurring in winemaking, and to obtain final products that can be submitted to sensory evaluation by panel of experts.

## Author contributions

Fundamental contributions to the conception and design of the work (GB, FG), acquisition, analysis and interpretation of data (GB, MT, CV); drafting the work and revising it critically for intellectual content (GB, FG, GM). All authors approved the final version of the manuscript to be submitted for publication and agreed to be accountable for all aspects of the work in ensuring that questions related to the accuracy and integrity of any part of the work are appropriately investigated and resolved.

### Conflict of interest statement

The authors declare that the research was conducted in the absence of any commercial or financial relationships that could be construed as a potential conflict of interest.
